# Heat-stable enterotoxin induced apoptosis in small intestine epithelial cells via mitochondrial oxidative phosphorylation pathway

**DOI:** 10.3389/fvets.2025.1545696

**Published:** 2025-05-06

**Authors:** Meijia Hou, Wei Yang, Nian Liu, Simeng Sun, Qizheng Feng, Bo Shi, Jiali Liu, Xiumei Dong

**Affiliations:** ^1^Veterinary Medicine College, Northeast Agricultural University, Harbin, China; ^2^Heilongjiang Key Laboratory for Animal Disease Control and Pharmaceutical Development, College of Veterinary Medicine, Northeast Agricultural University, Harbin, China

**Keywords:** STa, intestinal epithelium, mitochondria, apoptosis, enterotoxigenic *Escherichia coli* (ETEC)

## Abstract

Newborn piglet diarrhea caused by enterotoxigenic *Escherichia coli* (ETEC) causes serious economic losses in the swine industry worldwide. Heat-stable enterotoxins (STa) secreted by ETEC can damage the intestine, resulting in villus atrophy and shedding, which is the main cause of diarrhea in newborn piglets; however, the mechanism is not clear. This experiment was conducted *in vivo* (three-day-old suckling mice) and *in vitro* (IPEC-J2 cells) to explore the effect of STa on the intestinal epithelium by comparing the differences after infection with STa toxin-secreting *E. coli* O142 or STa-knockout *E. coli* O142ΔestA. The results showed that STa caused diarrhea, small intestinal edema, atrophy and rupture of small intestinal villi, and death in a dose-dependent manner in mice, and downregulated oxidative phosphorylation (OXPHOS) in IPEC-J2 cells. Activation of the mitochondria-mediated cell apoptosis pathway through excessive reactive oxygen species (ROS) production induces injury of the small intestinal villi, leading to diarrhea in piglets.

## 1 Introduction

Enterotoxigenic *Escherichia coli* (ETEC) is one of the main causes of diarrhea in neonatal and weaned piglets (1), resulting in great economic losses to the swine industry due to reduced growth performance of piglets and increased morbidity and mortality (2, 3). ETEC-induced diarrhea depends on adhesion to the epithelial villi of the small intestine and enterotoxin secretion (4, 5). Enterotoxins belong to two major classes, heat-labile enterotoxins (LT) and heat-stable enterotoxins (ST). Heat-resistant enterotoxins can be divided into STa and STb according to their solubility in methanol (6), both of which are composed of a single peptide chain (5). STa is synthesized as a precursor molecule of 72 amino acids, referred to as pre-pro-STa, which is cleaved in the periplasmic space to yield a mature peptide of 18 or 19 amino acids, with a molecular weight of approximately 2 kDa (5). When acting on intestinal mucosal epithelial cells, ST acts on the membrane surface or in the membrane and does not enter the cytoplasm. STa-mediated diarrhea begins with the binding of STa to its receptor, guanylate cyclase C (GC-C). The binding of STa to the extracellular domain of GC-C and the activation of the intracellular catalytic domain of GC-C results in the hydrolysis of GTP and cellular accumulation of cGMP (5).

Diarrhea mediated by STa has been shown to be accompanied by histological damage to the intestine, characterized by shortening and atrophy of the villi, a reduced ratio of small intestinal villi height to crypt depth, and a reduced villi surface (7). This damage leads to impaired absorption in the small intestine. Damage to villous epithelial cells of the small intestine, which absorb amino acids, glucose, and inorganic salts from the digestive tract into the bloodstream, affects the absorption of these nutrients (8). However, there are few reports on the specific molecular mechanisms by which STa leads to small intestinal epithelial cell injury and intestinal villus shedding.

Apoptosis is a type of programmed cell death that involves two classical pathways: the exogenous death receptor pathway and the endogenous mitochondrial pathway, of which the mitochondrial pathway is dominant. The ability of enterotoxins to induce apoptosis is well documented (9). STp inhibits cell proliferation and induces apoptosis of mice intestinal stem cells by down-regulating the Wnt/β-catenin signal transduction pathway (10). LT induces intestinal epithelial cell apoptosis through the endoplasmic reticulum pathway by causing upregulation of ROS. The ROS inhibitor, NAC, alleviates apoptosis caused by LT (11). Heat-labile enterotoxin IIc (LT-IIc) induces apoptosis in breast cancer cells MDA-MB-231 by increasing caspase-3/7 activity (12). *Clostridium perfringens* enterotoxin (CPE) causes caspase-3-dependent apoptosis in the human intestinal epithelial cell line, Caco-2 (13). The cytotoxic enterotoxin of *Aeromonas* (Act) may induce apoptosis in the human intestinal epithelial cell line, HT-29, by binding to protein-binding partners, including galectin-3 and the SNARE complex scaffolding protein synaptosomal-associated protein 23 (SNAP23) (14). We found that STa inhibits the OXPHOS pathway in IPEC-J2 cells. OXPHOS is the process by which mitochondria transfer electrons through the respiratory chain to generate adenosine triphosphate (ATP). Therefore, STa leads to apoptosis and small intestinal villus damage through this pathway, causing diarrhea. Although STa is the main toxin associated with diarrhea in neonatal pigs (5, 6), the molecular mechanism underlying STa interactions with epithelial cells remains relatively unknown. In this study, IPEC-J2 cells and three-day-old suckling mice were infected with STa-bearing wild-type and STa knockout recombinant bacteria to explore the molecular mechanism by which the STa toxin induces apoptosis and causes cell damage.

## 2 Materials and methods

### 2.1 Experimental design

To investigate the mechanistic effects of STa enterotoxin on small intestinal epithelial cells, we adopted a dual experimental strategy encompassing *in vitro* and *in vivo* models.

For the *in vitro* studies, cytotoxicity assessment of STa-treated epithelial cells was first performed. RNA sequencing (RNA-seq) was subsequently employed to identify differentially expressed genes (DEGs) and conduct pathway enrichment analysis. The transcriptomic findings were further validated through complementary biochemical assays.

In the *in vivo* experiments, 3-day-old neonatal mice were orally administered bacterial suspensions containing either wild-type *E. coli* O142 (STa-producing) or its isogenic mutant *E. coli* O142ΔestA (STa-deficient) at graded concentrations. The optimal bacterial inoculum for inducing enterotoxicity was determined by monitoring clinical manifestations and histopathological alterations in intestinal tissues via hematoxylin–eosin (H&E) staining. Crucially, the molecular mechanisms identified *in vitro* were functionally corroborated in the *in vivo* infection model.

### 2.2 Bacterial strains and growth conditions

The ETEC strain of *E. coli* O142 (serotype O142; ST^+^) was collected and an *E. coli* O142 mutant lacking STa expression (*E. coli* O142ΔestA) was constructed in a previous study (15). Both strains were used to infect IPEC-J2 cells. The frozen bacteria were resuspended in LB broth and inoculated onto Luria-Bertani (LB) plates. Single colonies were then selected and placed in 100 mL LB medium at 37°C and 170 rpm. Bacteria were collected in the logarithmic growth phase and resuspended in Dulbecco’s modified Eagle’s medium (DMEM) without serum and antibiotics. The concentration of the bacteria was adjusted, and the cells were infected in the next step. The colony-forming units (CFU) of the bacteria were determined by plotting growth curves through live bacterial counting.

### 2.3 Culture of epithelial cells and animals

The porcine jejunal intestinal cell line, IPEC-J2, is a non-transformed epithelial cell line that originates from a neonatal unsuckled piglet. It was isolated in 1989 by Helen Berschneider (16). IPEC-J2 cells were cultured in DMEM (Hyclone, United States) supplemented with 10% Sijiqing fetal bovine serum (Zhejiang Tianhang Biotechnology Co., Ltd., Zhejiang, China) and 1% Penicillin–Streptomycin Solution (100 U/mL, 0.1 μg/mL, respectively) (Beyotime Biotechnology, China). The cells were maintained in 25 cm^2^ cell culture flasks or 6/24/96-well cell plates (5% CO_2_, 37°C) (Corning, NY) in a humidified atmosphere and cultured for 24 h before receiving various treatments.

All procedures used in this study were approved by the Institutional Animal Care and Use Committee (IACUC) of Northeast Agricultural University (NEAUEC2021 03 42). Twenty male and female mice were randomly grouped and placed in cages for mating. The three-day-old offspring were randomly selected for oral gavage of *E. coli* O142 or O142Δ*estA*. The negative control cells were treated with phosphate-buffered saline (PBS). All mice were housed in an environmentally controlled room. Food and water were freely provided using traditional feeding methods. No parent mice died during feeding and no suckling mice died during gavage. Eight hours after intragastric administration, the suckling mice were euthanized and the small intestinal tissue was harvested for subsequent experiments.

### 2.4 Determination of lactate dehydrogenase activity

The lactate dehydrogenase (LDH) cytotoxicity assay kit (Beyotime Biotechnology, China) was used to evaluate the cytotoxicity induced by STa. When the cells in the 96-well plates had grown to 80–90%, they were washed three times with sterile PBS and bacteria resuspended in serum-free and antibiotic-free DMEM were added. The negative control contained only DMEM. *E. coli* O142 and O142Δ*estA* with different multiplicity of infection (MOI) were used to infect IPEC-J2 cells, and the LDH released from the cells at different times was measured. We infected IPEC-J2 cells with ETEC at an MOI of 25, 50, 75, 100, 150, and 200. The CFU quantities were measured and normalized according to minor differences in the bacterial inoculations. After incubation for various times (1, 2, 4, and 6 h), the operations and result calculations were strictly performed according to the manufacturer’s protocol to detect LDH secreted by cells.

### 2.5 Cell apoptosis detection using Annexin V-FITC and propidium iodine staining assays

IPEC-J2 cells were grown in 6-well tissue culture plates and infected with *E. coli* O142 and *E. coli* O142Δ*estA* at an MOI of 100. The negative control cells were infected with the same volume of DMEM. Cells were harvested and washed with cold PBS. Apoptosis was determined using an Annexin V-FITC/PI cell apoptosis detection kit (Wanleibio Co., Ltd., Shengyang, Liaoning, China), according to the manufacturer’s instructions. The cells were suspended in 500 μL binding buffer, and then incubated with Annexin V-FITC and propidium iodine (PI) at room temperature for 15 min in the dark, followed by analysis using a flow cytometer.

### 2.6 Transmission electron microscopy

For electron microscopy analysis, IPEC-J2 cells were infected with *E. coli* O142 and *E. coli* O142Δ*estA* at an MOI of 100. The negative control cells were incubated with the same volume of DMEM. After infection, at least 5 × 10^6^ cells were collected from each group of samples and fixed with 2.5% glutaraldehyde at 4°C overnight, followed by washing with 0.1 M PBS three times and 1% osmic acid fixation at 4°C for 2 h. Thereafter, cells were washed with PBS and underwent acetone gradient dehydration, resin embedding, sectioning, and negative staining. The cells were observed using an H-7650 transmission electron microscope (Hitachi, Tokyo, Japan).

### 2.7 Hematoxylin-eosin staining

Three-day-old suckling mice were orally given 3 × 10^7^, 3 × 10^8^, 7.5 × 10^8^, 1.5 × 10^9^, 2.25 × 10^9^, and 3 × 10^9^ CFU *E. coli* O142 bacterial suspension, and 3 × 10^9^ CFU *E. coli* O142Δ*estA* bacterial suspension, separately. Negative control suckling mice were treated with the same volume of PBS. Nine hours later, the small intestinal tissue was fixed in 4% paraformaldehyde for 48 h and embedded in paraffin blocks to prepare sections. The sections were stained using an hematoxylin-eosin (HE) Staining Kit (Beijing Leagene Biotechnology Co., Ltd., Beijing, China) and visualized using Lionheart LX Automated Microscope (BioTek, Vermont, United States), the images were obtained by Gene5 3.05 Imager.

### 2.8 RNA-seq transcriptomic assay

IPEC-J2 cells were grown in 25 cm^2^ cell culture flasks, infected with ETEC strains at an MOI of 100, and cultured in cell incubators for 2 h. Negative control cells were treated with the same dose of DMEM. Three replicates were used for each group. After incubation, the cells were washed three times with cold PBS and lysed with TRIzol (Invitrogen). Total RNA from IPEC-J2 cells (1 μg) was used as starting material for deep sequencing using NEBNext^®^ UltraTM RNA Library Prep Kit for Illumina^®^ (NEB, United States). Briefly, mRNA was purified using oligo-dT beads, fragmented with divalent cations, subjected to heat-catalyzed hydrolysis, and used as a template for first- and second-strand complementary deoxyribonucleic acid (cDNA) synthesis with random primers. The cDNA 3′ ends were adenylated, followed by adaptor ligation and PCR to enrich DNA fragments. The cDNA libraries were quantified using a Qubit2.0 Fluorometer and an Agilent 2100 bioanalyzer. The cDNA libraries were pooled at a final concentration of 1.5 ng/μL. Paired-end sequencing was performed by Shanghai Novogene Co., Ltd. (Beijing, China) using an Illumina novaseq6000 (Illumina, CA, United States). Differential expression analysis was performed using DESeq2. Differential gene expression enrichment was analyzed using Gene Ontology (GO) tools.^1^ Differentially expressed genes (DEGs) were considered significantly enriched at *p*-values <0.05. The Profiler R package was used to test the statistical enrichment of genes in the Kyoto Encyclopedia of Genes and Genomes (KEGG) pathway.^2^

### 2.9 RT-qPCR analysis of genes at mRNA levels

IPEC-J2 cells were grown in 24-well plates, infected with ETEC strains at an MOI of 100, and cultured in a cell incubator for 2 h. Negative control cells were treated with the same dose of DMEM. Total RNA was extracted using an RNAprep Pure Cell/Bacteria Kit (Tiangen Biotech, Beijing, China). RNA was quantified using a Nano-300 (Hangzhou Allsheng Instruments Co., Ltd., Zhejiang, China), and 1.0 μg RNA was reverse transcribed with 5× FastKing-RT SuperMix (Tiangen Biotech, Beijing, China). The RT-qPCR was performed using a Light Cycler^®^ 480 System (Roche, Basel, Switzerland) and 2× SYBR Green qPCR Master Mix (Bimake, Shanghai, China) according to the reaction system and conditions (Table 1). Five of the most significant DEGs between the *E. coli* O142-infected and *E. coli* O142Δ*estA* groups according to RNA sequencing data (log_2_(fold change) >±1.0; *p*-value <0.05) were selected for RT-qPCR. Three replicates per sample were performed for each RT-qPCR assay and β-actin was used as the housekeeping gene for normalization. Specific primers for RT-qPCR were designed using the assembled RNA-Seq sequence data (Table 2). The gene’s relative abundance of mRNA was determined using the comparative threshold cycle (Ct) method by 2^−ΔΔCT^: ΔCt (test) = Ct (target gene, test) − Ct (β-actin, test), ΔCt (control) = Ct (target gene, control) − Ct (β-actin, control), ΔΔCT = ΔCt (test) − ΔCt (control). Only one peak was observed for each PCR product in the melting curve analysis.

**Table 1:** RT-qPCR reaction system and conditions.

Composition	Dosage per reaction (μL)	Application setting	Temperature (°C)	Time (s)	Cycle
2× SYBR green qPCR master mix	25	Hot-start DNA polymerase activation	95	30	
cDNA template	100 ng	PCR	95	15	40
Upstream primer (10 μM)	2.5		55	30	
Downstream primer (10 μM)	2.5		72	30	
ddH_2_O	Add to 20	Melt Curve	95	15	1
Total reaction volume	20		60	60	
			95	15	

**Table 2:** The primers used in the present study for IPEC-J2.

Target gene	GenBank ID	Primer sequence (5′–3′)
β-actin	XM_003124280.5	Forward: CAGGTCATCACCATCGGCAACG
		Reverse: GACAGCACCGTGTTGGCGTAGAGGT
ND2	NC_000845	Forward: TGCCTCCACTATCAGGATTTAT
		Reverse: ATTGTCATTTTATTTTTATGTTGTT
COXII	NC_000845	Forward: CGCCACTTCACCCATCATA
		Reverse: GCTAGTGTGTGTCAGTTTTGTTGT
ATP6	NC_000845	Forward: ATTTGCCTCTTTTATTGCCC
		Reverse: TATTATTTGTTTGGATGTTAGTTGG
CYTB	NC_000845	Forward: AAAATTATCAACAACGCATTCATTG
		Reverse: TAGGATTTGCAAGATTAGGCAGAT

Three-day-old suckling mice were orally administered 3 × 10^9^ CFU of the bacterial suspension. Nine hours later, the jejunal tissue was collected. Negative control suckling mice were treated with the same dose of PBS. Total RNA was extracted using the RNAprep Pure Tissue Kit (Tiangen Biotech, Beijing, China). RT-qPCR was performed as previously described. Specific primers for RT-qPCR were designed using assembled RNA-Seq sequence data (Table 3).

**Table 3:** The primers used in the present study for mice.

Target gene	GenBank ID	Primer sequence (5′–3′)
β-actin	NM_007393.5	Forward: TTTCCAGCCTTCCTTCTTG
		Reverse: ACAGCACTGTGTTGGCATAGA
ND2	NC_005089	Forward: TATCACCCTTGCCATCATCT
		Reverse: GCTGCTTCAGTTGATCGTG
COXII	NC_005089	Forward: CAAGCACAATAGATGCACAAG
		Reverse: TATGGTTTTAACGGTTAATACG
ATP6	NC_005089	Forward: CTATTTGCCTCATTCATTACCC
		Reverse: GGAAAGAATGGAGACGGTTG
CYTB	NC_005089	Forward: AATAGTCCAAATCATTACAGGTCTT
		Reverse: TTTGCGTGTATATATCGGATTAGT

### 2.10 Western blot

IPEC-J2 cells were grown in 25 cm^2^ cell flasks, infected with ETEC strains at an MOI of 100, and cultured in a cell incubator for 2 h. Negative control cells were treated with the same volume of DMEM. The medium in each flask was removed and cells were washed three times with sterile PBS. Total protein was extracted using 100 μL Radio Immunoprecipitation Assay (RIPA) lysis buffer (Beyotime Biotechnology, China) containing 1 μL phenylmethanesulfonyl fluoride (PMSF, Beyotime Biotechnology, China). The cells were incubated on ice for 1 min, scraped off with a cell scraper, and aspirated into an EP tube using a micropipette. Tubes were incubated at 4°C for 15 min, and total protein was isolated by centrifugation (12,000 rpm, 10 min, 4°C) in a high-speed low-temperature centrifuge (Thermo Scientific). The supernatant was transferred to a new tube to obtain total protein.

Cytoplasmic and mitochondrial protein extraction from IPEC-J2 cells: at least 5 × 10^7^ cells were collected and added to the mitochondrial extraction reagent. After centrifugation at 3,000 rpm for 15 min at 4°C, the supernatant was collected and centrifuged at 20,000 rpm for 20 min at 4°C. The supernatant was collected as the cytoplasmic protein. The precipitate was added to RIPA lysis buffer, sonicated in an ice-water bath, centrifuged at 10,000 rpm for 10 min, and the supernatant was collected as the mitochondrial protein.

Three-day-old suckling mice were orally administered 3 × 10^9^ CFU of the bacterial suspension. Nine hours later, the jejunum tissue was ground using the Bioprep-24 Homogenizer tissue grinding instrument (Hangzhou Allsheng Instruments Co., Ltd., Zhejiang, China) in 1 mL RIPA containing 10 μL PMSF. Tissue homogenate was sonicated followed by centrifugation at 12,000 rpm for 10 min at 4°C. The supernatant was collected as total protein. Small intestine tissue from suckling mice supplemented with mitochondrial extraction reagent was ground in a grinding tube, and the tissue homogenate was extracted according to the IPEC-J2 cell protocol. An Enhanced BCA Protein Assay Kit (Beyotime Biotechnology) was used to determine protein concentration. All samples were mixed with 5× sodium dodecyl sulfate-polyacrylamide gel electrophoresis (SDS-PAGE) Sample Loading Buffer (Beyotime Biotechnology) and boiled for 10 min. The protein samples were stored at −20°C.

Western blotting was used to detect the effect of STa on protein expression in the intestinal epithelial cells (34). An equal amount of 60 μg protein was loaded and separated using 12% SDS-PAGE. Proteins were transferred to a nitrocellulose (NC) membrane. Membranes were blocked with Tris-buffered saline-0.1% Tween 20 (TBST) containing 5% fat-free milk powder at room temperature (RT) on a horizontal shaker for 2 h. The membranes were then rinsed thrice with TBST for 10 min. The membranes were incubated overnight with diluted primary antibodies. Thereafter, membranes were washed in TBST three times for 10 min, followed by incubation with the corresponding HRP-linked secondary antibodies (dilution, 1:1,000) for 1 h at RT, then washed with TBST as described above. Protein bands were visualized using an electrochemiluminescence (ECL) detection system (Wanleibio Co., Ltd., Shengyang, Liaoning, China) and quantitatively analyzed using a Quantity One image densitometer (Clinx Science Instruments Co., Ltd., Shanghai, China). The blots shown in the figures are representative of at least three biological replicates and were quantified using ImageJ software. The primary and secondary antibodies were diluted in TBST at different concentrations (Table 4). The β-actin content was analyzed as the loading control of the total cell protein. Mitochondrial protein levels were standardized by comparison with voltage dependent anion channel protein 1 (VDAC1) levels.

**Table 4:** The antibodies used in the present study.

Antibody name	Dilution ratio
Anti-Bcl-2 antibody	1:500
Anti-Bax antibody	1:500
Anti-COX II antibody	1:500
Anti-Cyt-c antibody	1:500
Anti-caspase-9 antibody	1:500
Anti-caspase-3 antibody	1:500
Anti-cleaved caspase-9 antibody	1:500
Anti-cleaved caspase-3 antibody	1:500
Anti-MT-ND2 antibody	1:500
Anti-NDUFS8 antibody	1:500
Anti-β-actin antibody	1:1,000
Anti-VDAC1 antibody	1:500
Goat anti-rabbit antibody of IgG-HRP	1:8,000
Goat anti-mouse antibody of IgG-HRP	1:8,000

Rabbit polyclonal antibodies against B-cell lymphoma 2 (Bcl-2), Bcl-2 associated X (Bax), cytochrome C (Cyt c), caspase-9, caspase-3, cleaved caspase-9, cleaved caspase-3, and VDAC1 were purchased from Wanleibio (Shenyang, China). Rabbit polyclonal antibodies against COX-II and MT-ND2 were purchased from ImmunoWay Biotechnology (Plano, TX, United States). β-actin was purchased from Abmart (Shanghai, China). Goat anti-rabbit IgG-horseradish peroxidase antibody was obtained from Cell Signaling Technology (Danvers, MA, United States). Goat anti-rabbit IgG-horseradish peroxidase and goat anti-rabbit IgG-horseradish peroxidase antibodies were purchased from Nachuan Biotechnology (Harbin, Heilongjiang, China).

### 2.11 ROS measurement

The ROS assay kit was purchased from Beyotime Biotech Co., Ltd. (Nantong, China) and was used to evaluate the generation of intracellular ROS with the fluorescent probe, 2′,7′-dichlorofluorescin diacetate (DCFH-DA). IPEC-J2 cells were seeded in a 24-well cell culture plate, infected with ETEC strains at an MOI of 100, and cultured in a cell incubator for 2 h. Negative control cells were treated with the same dose of DMEM. After treatment with *E. coli* O142 or *E. coli* O142Δ*estA*, the cells were incubated with DCFH-DA at 37°C for 30 min and then washed with PBS. Cells were observed using a Lionheart LX Automated Microscope (Biotek, Winooski, Vermont, United States).

### 2.12 Superoxide dismutase, catalase, glutathione, 8-OHDG (8-hydroxy-2-deoxyguanosine), and mitochondrial respiratory chain complex I activity

IPEC-J2 cells were collected, and the superoxide dismutase (SOD)/catalase (CAT)/glutathione (GSH)/8-OHDG (8-hydroxy-2-deoxyguanosine)/adenosine triphosphate (ATP) of different treatment groups was determined using ELISAs according to the kit instructions (Nanjing Jiancheng Bioengineering Institute, Nanjing, China). Mitochondrial respiratory chain complex I activity was determined according to the manufacturer’s instructions (Beijing Solarbio Science & Technology Co., Ltd., Beijing, China).

### 2.13 Determination of mitochondrial membrane potential

A mitochondrial membrane potential Detection Kit (JC-1) (Beijing Solarbio Science & Technology Co., Ltd., Beijing, China) was used to determine the MMP of IPEC-J2 cells. JC-1 is an ideal fluorescent probe widely used to detect mitochondrial membrane potential (ΔΨm, MMP). JC-1 aggregates in the mitochondrial matrix to form polymers and produces red fluorescence when the mitochondrial membrane potential is high. JC-1 cannot aggregate in the mitochondrial matrix with a low mitochondrial membrane potential, but instead emits green fluorescence as a monomer. A decrease of mitochondrial membrane potential is a landmark event in the early stages of apoptosis and can be easily detected by the transition of JC-1 from red to green fluorescence. IPEC-J2 cells were collected after ETEC treatment, incubated with JC-1 at 37°C for 20 min, washed twice with JC-1 staining buffer, and observed under a Lionheart LX Automated Microscope (Biotek).

### 2.14 Immunofluorescence

IPEC-J2 cells were grown in a 24-well cell plate at 37°C with 5% CO_2_. Cells were cultured in a monolayer, inoculated with *E. coli* O142Δ or *E. coli* O142Δ*estA* at a 1:100 MOI, and further cultured for 2 h. The supernatant was discarded and the cells were washed with PBS three times (5 min/wash). The cells in the control group were treated with the same volume of DMEM. Two hours post infection, IPEC-J2 cells were fixed with 4% cold paraformaldehyde for 30 min at room temperature. After three PBS washes (3 min/wash), IPEC-J2 cells were permeabilized with 0.2% Triton-100 in PBS for 10 min at room temperature. Then cells were washed with PBS buffer, and blocked with 5% BSA in PBS for 30 min at 37°C. After three PBS washes, IPEC-J2 cells were incubated with a 1:500-diluted Cyt c rabbit polyclonal antibody at 4°C overnight, and treated with a 1:100 dilution of FITC-labeled goat anti-rabbit IgG for 1 h. Thereafter, cells were stained with a 1:4-diluted 4′,6-diamidino-2-phenylindole dihydrochloride (DAPI) solution for 15 min at room temperature. After three rinses, the samples were covered with PBS and visualized using a microscope and software as described above.

### 2.15 Immunohistochemistry

Three-day-old suckling mice were orally given 3 × 10^9^ CFU *E. coli* O142 or *E. coli* O142Δ*estA* bacterial suspension, separately. Negative control suckling mice were treated with the same volume of PBS. The paraffin sections are described in section 2.7. Immunohistochemistry was performed using a DAB Horseradish Peroxidase Color Development Kit (Beijing Leagene Biotechnology Co., Ltd., Beijing, China) and a Streptavidin-Peroxidase Immunohistochemical staining kit (Beijing Biosynthesis Biotechnology Co., Ltd., Beijing, China). The sections were visualized using Lionheart LX Automated Microscope (BioTek, Vermont, United States), the images were obtained by Gene5 3.05 Imager.

### 2.16 Statistical analysis

Experiments were conducted in triplicate on at least three separate occasions with similar results. The data were analyzed using one-way analysis of variance (ANOVA followed by Tukey’s multiple comparisons), using Graph Pad Prism software (version 7.0, Graph Pad Software Inc., San Diego, CA, United States), and reported as the mean ± standard error of the mean. *p*-values of 0.01 to 0.05, 0.001 to 0.01, 0.0001 to 0.001, and ≤0.0001 were considered significant and represented with *, **, ***, and ****.

## 3 Results

### 3.1 STa infection induces apoptosis in IPEC-J2 cells and small intestinal villi injury in mice

**Figure 1:**
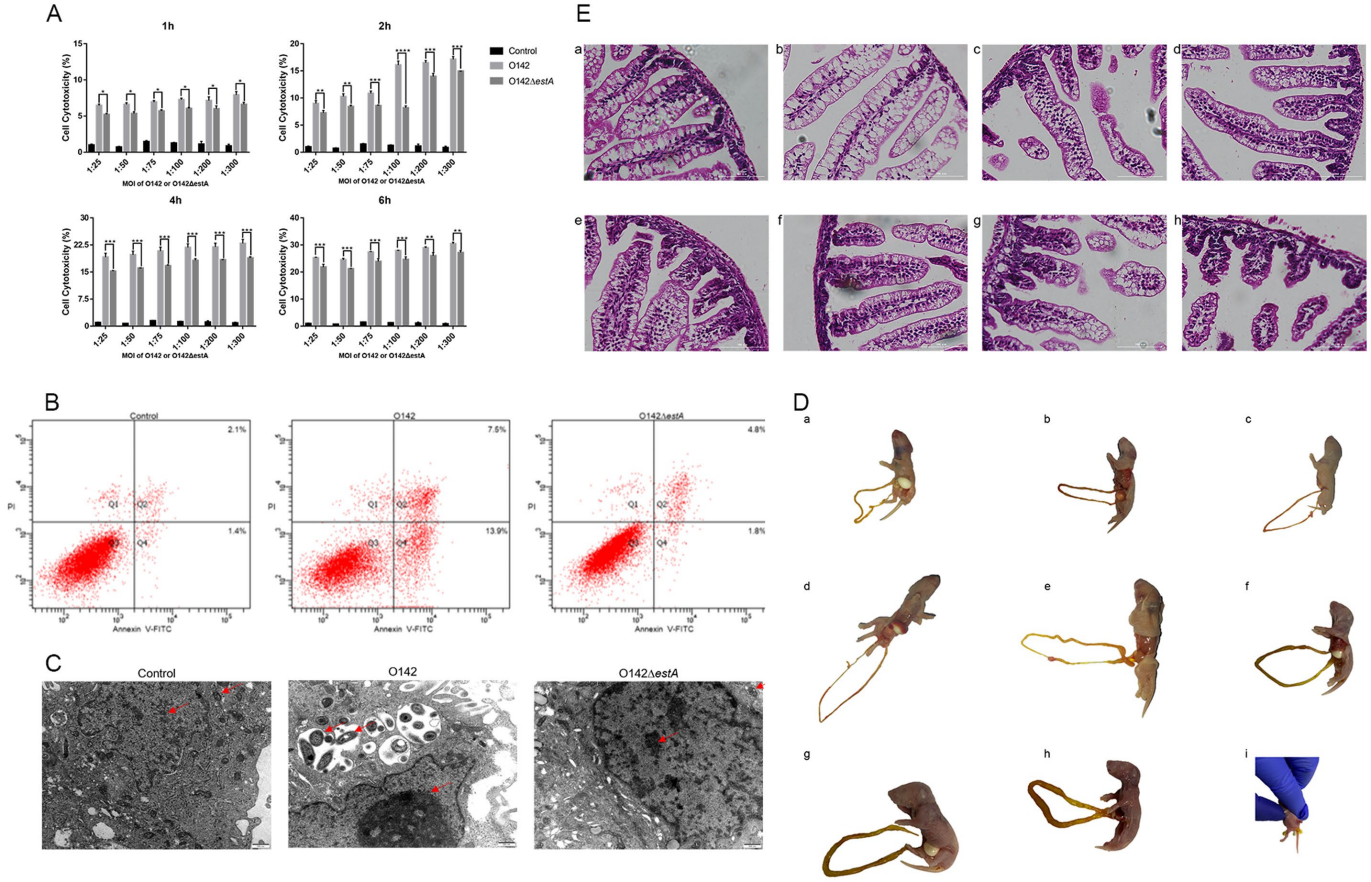
Toxicity of STa on IPEC-J2 cells and suckling mice. **(A)** Cell cytotoxicity analysis by LDH assay. *E. coli* O142 or *E. coli* O142Δ*estA* were applied to IPEC-J2 cells for MOI of 1:25, 1:50, 1:75, 1:100, 1:200, 1:300, and for 1-, 2-, 4-, or 6-h. After infection, aliquots were collected from the supernatants and assessed for cell cytotoxicity by ELISA. As shown, STa intoxication significantly increases the amount of LDH measured in supernatants. This data shows that STa induces epithelial cell apoptosis. (Error bars is on behalf of mean ± SEM (standard error of mean), ^*^p < 0.05, ^**^p < 0.01, ^***^p < 0.001, and ^****^p < 0.0001, one-way ANOVA). **(B)** Cell apoptosis analysis by flow cytometry with Annexin V-FITC/PI cell labeling. IPEC-J2 cells were collected after, respectively, infected with *E. coli* O142 and *E. coli* O142Δ*estA* and were dual-labeled with Annexin V-FITC and PI and analyzed by flow cytometry. Lower left quadrants represent intact cells (Annexin V-FITC and PI double negative, Annexin V-FITC^−^/PI^−^); lower right quadrants represent early apoptotic cells (Annexin V-FITC positive and PI negative, Annexin V^+^/PI^−^); upper right quadrants indicate late apoptotic cells (Annexin V-FITC PI double positive, Annexin V^+^/PI^+^); upper left quadrants indicate necrotic cells (Annexin V-FITC PI double positive, Annexin V^+^/PI^+^). The percentage of cells were shown in each quadrant. Left is control group; middle is *E. coli* O142 infected group; right is *E. coli* O142Δ*estA* group. **(C)** The effect of STa on the morphology of IPEC-J2 cells (500 nm). **(D)** Observation of the state of suckling mice after perfusion with *E. coli* O142 or *E. coli* O142Δ*estA*. **(a)** PBS. **(b)** Fed with 3 × 10^9^ CFU *E. coli* O142Δ*estA* bacteria suspension. **(c)** Fed with 3 × 10^7^ CFU *E. coli* O142 bacteria suspension. **(d)** Fed with 3 × 10^8^ CFU *E. coli* O142 bacteria suspension. **(e)** Fed with 7.5 × 10^8^ CFU *E. coli* O142 bacteria suspension. **(f)** Fed with 1.5 × 10^9^ CFU *E. coli* O142 bacteria suspension. **(g)** Fed with 2.25 × 10^9^ CFU *E. coli* O142 bacteria suspension. **(h)** Fed with 3 × 10^9^ CFU *E. coli* C O142 bacteria suspension. **(i)** Diarrhea feces. **(E)** HE staining of small intestine of suckling mice after perfusion of *E. coli* O142 or *E. coli* O142Δ*estA* (100 μm). **(a)** PBS. **(b)** Fed with 3 × 10^9^ CFU *E. coli* O142Δ*estA* bacteria suspension. **(c)** Fed with 3 × 10^7^ CFU *E. coli* O142 bacteria suspension. **(d)** Fed with 3 × 10^8^ CFU *E. coli* O142 bacteria suspension. **(e)** Fed with 7.5 × 10^8^ CFU *E. coli* O142 bacteria suspension. **(f)** Fed with 1.5 × 10^9^ CFU *E. coli* O142 bacteria suspension. **(g)** Fed with 2.25 × 10^9^ CFU *E. coli* O142 bacteria suspension. **(h)** Fed with 3 × 10^9^ CFU *E. coli* O142 bacteria suspension.

The LDH released by the cells increases with the increase of the MOI and the prolongation of the infection time; therefore, it was used to test the cytotoxicity of STa in IPEC-J2 cells (Figure 1A). When the MOI was 1:100 and the infection time was 2 h, the difference between LDH released in *E. coli* O142- and *E. coli* O142Δ*estA*-infected cells was the largest. We used these conditions to investigate the effect of STa infection on apoptotic cell death using Annexin V and PI assays. The percentage of early apoptotic cells in IPEC-J2 cells infected with *E. coli* O142 was 13.9%, which was significantly higher than the number of cells infected with *E. coli* O142Δ*estA* (1.8%) (Figure 1B). *E. coli* O142 infection significantly increased apoptosis, and the percentage of apoptotic cells increased upon STa treatment, reaching 21.4%. Taken together, these data demonstrated that STa induces apoptosis in IPEC-J2 cells.

Transmission electron microscopy (TEM) was applied to detect the microstructure and more intuitively observe morphological changes to the cells after infection with *E. coli* O142 or *E. coli* O142Δ*estA*. IPEC-J2 cells in the control group displayed a normal structure, uniform distribution of nuclear chromatin, and normal cristae structure in the mitochondria (Figure 1C). After 2 h infection with *E. coli* O142 and *E. coli* O142Δ*estA*, respectively, IPEC-J2 cells showed varying degrees of injury. IPEC-J2 cells infected with ETEC O142 showed a typical apoptotic morphology, with large vacuoles appearing in the cytoplasm. The mitochondria were swollen, rounded, and vacuolated, and the mitochondrial cristae had disappeared. Many mitochondria appeared to be surrounded by vesicles, and chromatin aggregated near the edge of the nuclear envelope (Figure 1C). The chromatin of IPEC-J2 cells infected with *E. coli* O142Δ*estA* was slightly gathered, and the mitochondria showed a normal structure, clear bilayer, and cristae. Small vesicles were observed around the nucleus and holes were observed in the cytoplasm (Figure 1C).

Compared with the control group, mice given 3 × 10^8^ and 7.5 × 10^8^ and 3 × 10^9^ CFU *E. coli* O142, and 3 × 10^9^ CFU *E. coli* O142Δ*estA* bacterial suspension group showed no obvious symptoms. The skin of the mice was naturally pink, there was no diarrhea or feces in the anus, and the intestine was not filled after autopsy (Figures 1D,a–e). The mice given 1.5 × 10^9^ CFU *E. coli* O142 had slightly cyanotic skin and mild swelling of the small intestine (Figures 1D,f). The mice given 2.25 × 10^9^ CFU *E. coli* O142 occasionally had mild diarrhea, were cyanotic, and lacked energy. The small intestine exhibited mild swelling (Figures 1D,g). The mice administered 3 × 10^9^ CFU *E. coli* O142 were cyanotic, lethargic, and unwilling to move, with watery diarrheal stool at the anus, and intestinal swelling and filling were observed at autopsy (Figures 1D,h,i). Small-intestinal segments from each group were stained with HE to observe the shape of the small-intestinal villi. Compared with the control group, there were no significant changes in small intestinal villi of mice in the *E. coli* O142Δ*estA* group (Figures 1E,a,b). In the group that received various oral concentration of *E. coli* O142, with an increase in the bacterial suspension concentration, the small intestinal villi of the mice showed atrophy, fracture, and increased villus spacing (Figures 1E,c–h). These results show that STa damages the intestinal villi in a concentration-dependent manner.

### 3.2 Differential gene expression of IPEC-J2 cells and GO and KEGG enrichment analyses

**Figure 2:**
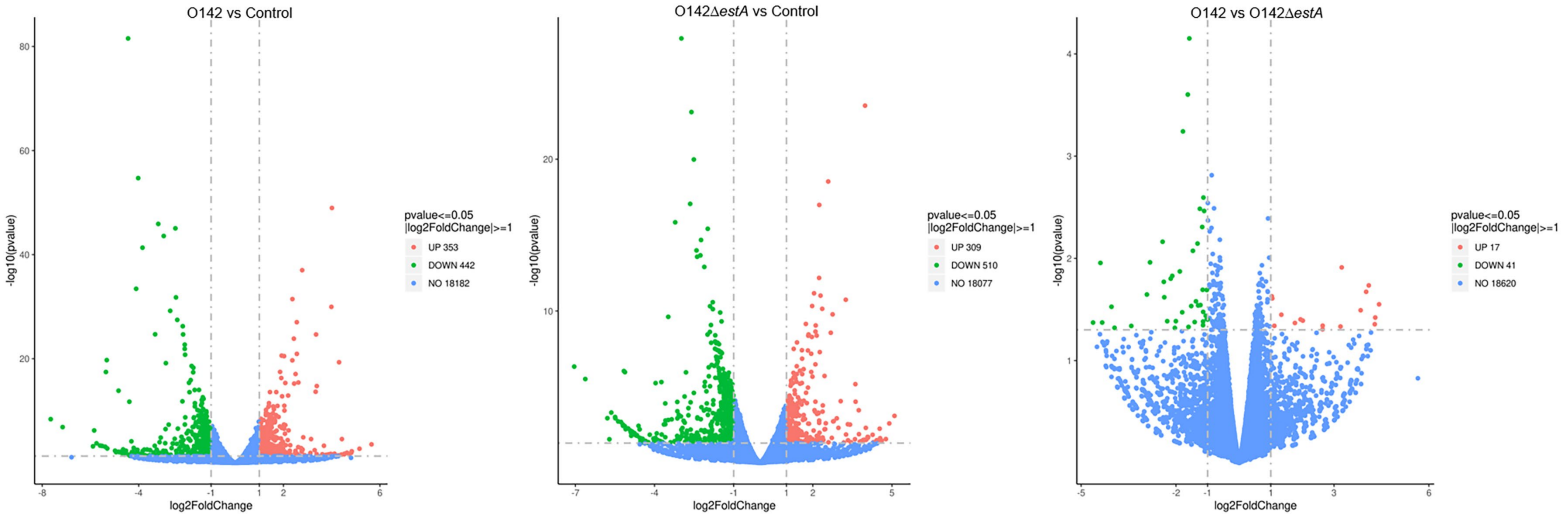
Volcanic map of DEGs distribution by RNA-seq. Volcano plots reflect overall gene expression. The abscissa represents log_2_(fold change), and the ordinate represents −log10 (*p*-value). log_2_FoldChange represents the ratio of gene expression levels in the treatment group and the control group, and then takes the logarithm with 2 as the base. *p*-value represents the *p*-value for the significance test. Each dot represents a gene, and the color is used to distinguish differentially expressed genes. Red indicates up-regulated genes, green indicates down-regulated genes, and gray dots indicates genes that are not differentially expressed. (*p*-value <0.05, |log_2_FoldChange| >0).

Differences in expression levels between the groups were considered significant after adjusting for multiple testing based on a *p*-value <0.05. We first filtered the genes based on *p*-values <0.05 and an absolute difference >± 2-fold (i.e., log_2_(fold change) >±1.0). In the volcano plot, red points indicate that the expression of genes increased, green points show the opposite, and blue points indicate no significant variation. In total, 18,977 DEGs were normalized and 795 significant DEGs were detected between the *E. coli* O142-infected and control groups, including 353 upregulated and 442 downregulated genes (Figure 2). The 18,896 DEGs were normalized and 819 significant DEGs were detected between the *E. coli* O142Δ*estA*-infected and control groups, including 309 upregulated and 510 downregulated genes (Figure 2). Moreover, 18,678 DEGs were normalized and 58 significant DEGs were detected between the *E. coli* O142-infected and *E. coli* O142Δ*estA*-infected groups, including 17 upregulated and 41 downregulated genes (Figure 2).

**Figure 3:**
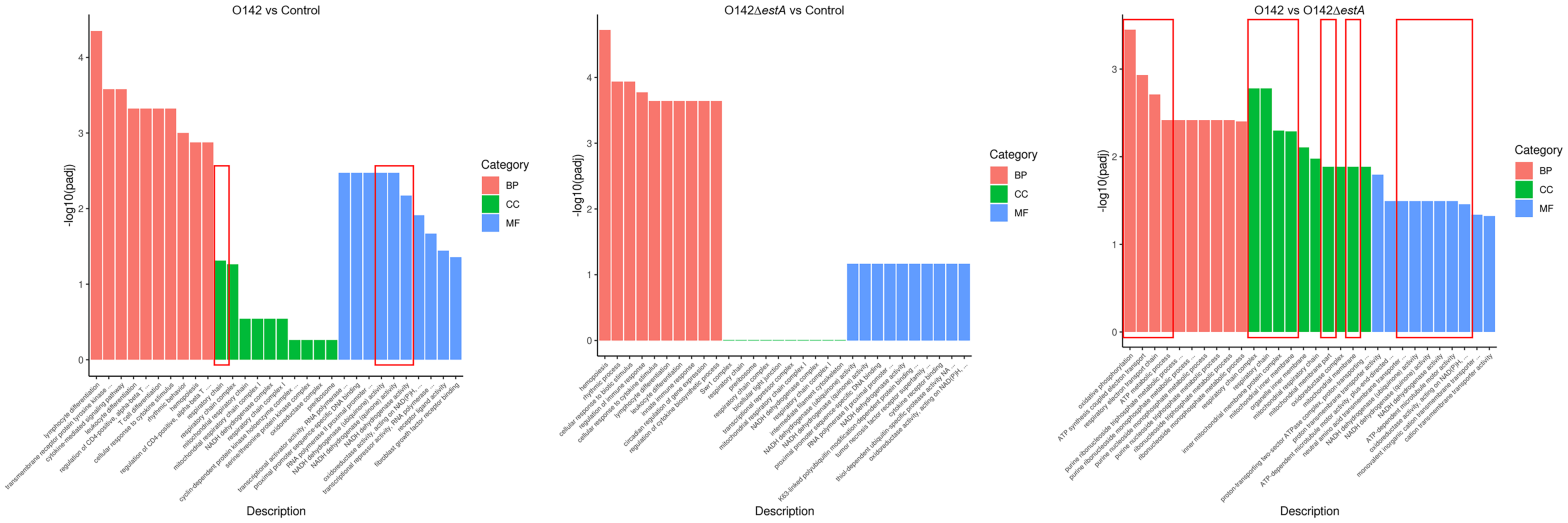
GO classification and functional annotation of MF, CC, and BP. The abscissa in the figure is GO term; the ordinate is the significance level of GO term enrichment, the higher the value, the more significant; the different colors represent the three GO subclasses of BP, CC, and MF, respectively.

GO is a comprehensive database that describes gene functions. GO function enrichment considers an adjusted *p*-value (*p*adj) of less than 0.05, which is the threshold for significant enrichment. GO functional cluster analysis divided the DEGs between control, *E. coli* O142-infected, and *E. coli* O142Δ*estA*-infected groups into three categories involving biological process (BP), cellular component (CC), and molecular function (MF) (Figure 3). From the GO enrichment analysis results, the most significant 10 terms in each function were selected to draw a histogram for display. The similarity in enrichment of GO functions between the *E. coli* O142- and *E. coli* O142Δ*estA*-infected groups was leukocyte and lymphocyte differentiation. The difference between *E. coli* O142- and *E. coli* O142Δ*estA*-infected groups enriched in GO function was that the *E. coli* O142-infected group was significantly enriched in term related to OXPHOS and mitochondrial respiratory chain complexes. It was speculated that STa mainly affected the OXPHOS function of IPEC-J2 cells and may cause apoptosis by inhibiting the expression of this gene, which needs to be further verified by KEGG pathway enrichment analysis.

**Figure 4:**
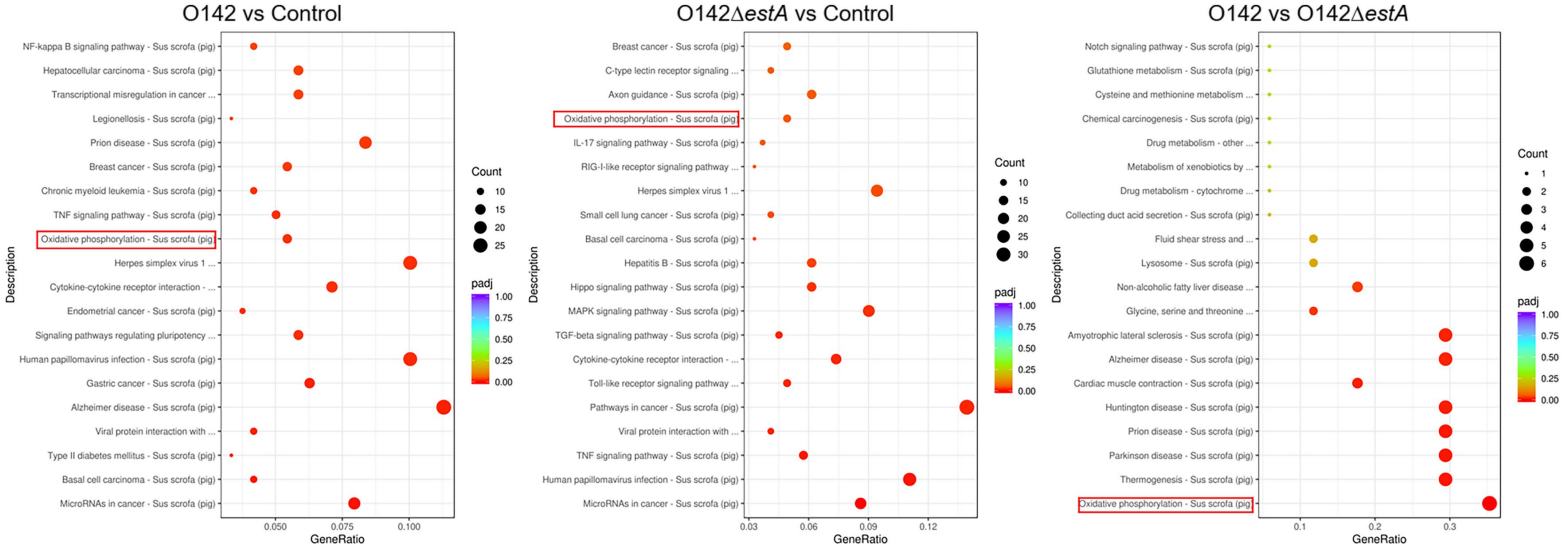
KEGG pathway classification and functional enrichment. The abscissa is the ratio of the number of differential genes annotated to the KEGG pathway to the total number of differential genes, the ordinate is the KEGG pathway, the size of the dots represents the number of genes annotated to the KEGG pathway, and the color from red to purple represents the significance of enrichment.

KEGG is a comprehensive database that integrates genome, chemistry, and system function information. KEGG pathway enrichment considers a *p*_adj_ of less than 0.05 as the threshold for significant enrichment. From the KEGG enrichment, the most significant 20 KEGG pathways were selected to draw a scatter plot. KEGG pathway analysis was performed to evaluate the biological and ontological significance of DEGs. The results showed that the DEGs were involved in a variety of biological activities. Both *E. coli* O142 and *E. coli* O142Δ*estA* infection altered gene expression levels of cancer-related cytokine-cytokine receptor interaction, TNF signaling pathway, and OXPHOS pathway (Figure 4). However, DEGs in the *E. coli* O142-infected group were more enriched, and the differences in DEGs were more significant.

**Figure 5:**
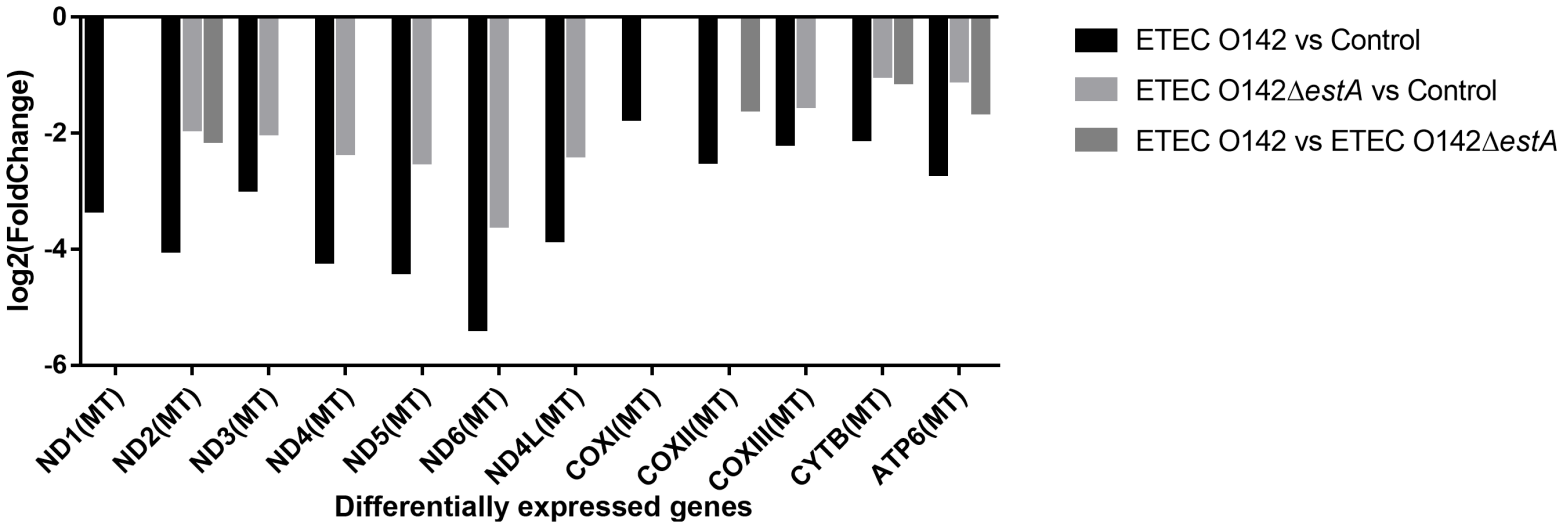
DEGs enrichment analysis between *E. coli* O142 and *E. coli* O142Δ*estA* group.

Compared with the *E. coli* O142Δ*estA*-infected group, NADH dehydrogenase (ND2), cytochrome C oxidase (COX2), cytochrome B (CYTB), and ATP synthase (ATP6) were significantly downregulated in *E. coli* O142-infected cells (Figure 5). These genes are related to OXPHOS phosphorylation.

### 3.3 Detection of OXPHOS related genes and proteins

**Figure 6:**
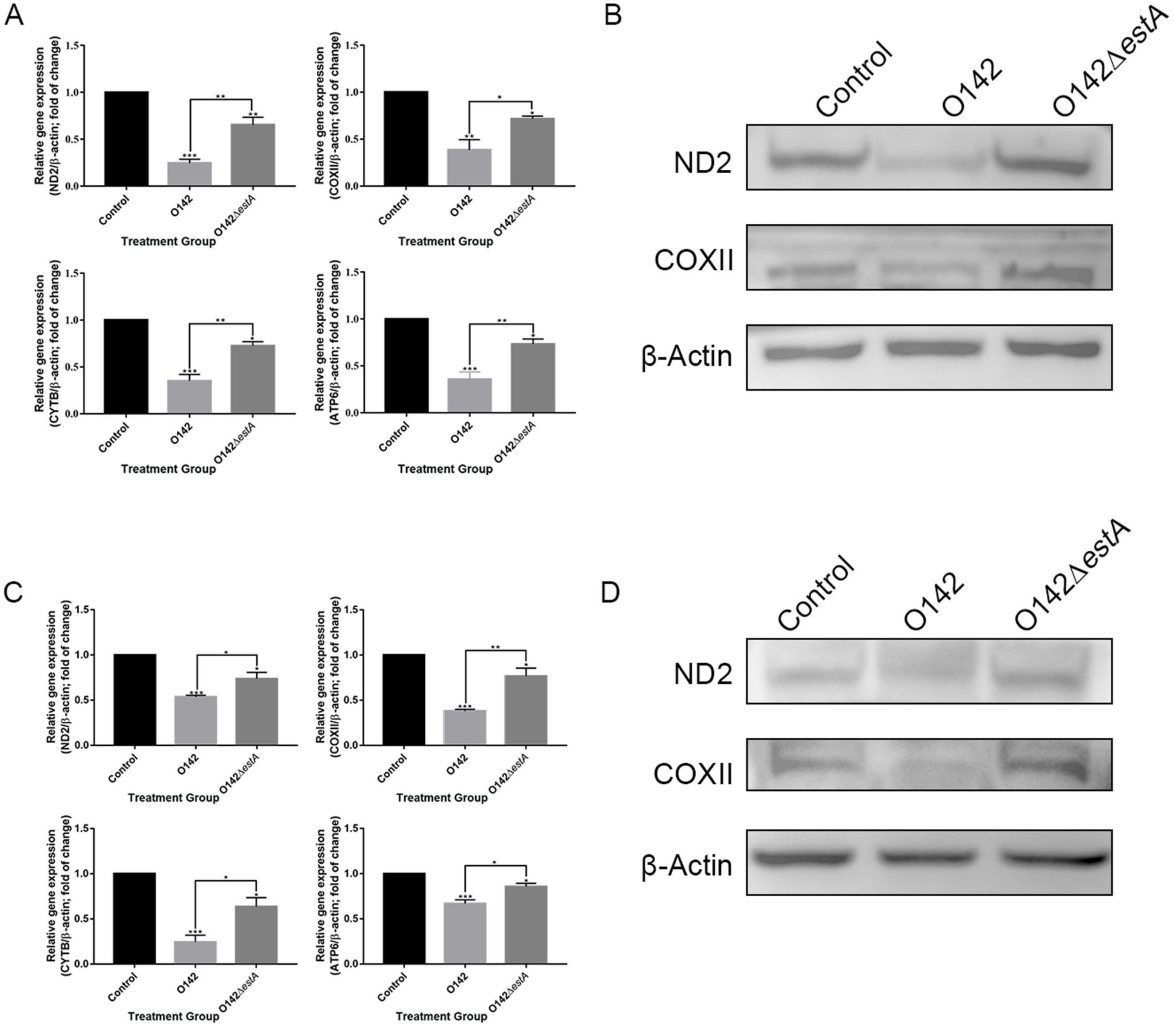
Mitochondrial respiratory chain gene expression levels. **(A)** mRNA expression by RT-qPCR of IPEC-J2. **(B)** The abundance of ND2 and COXII in IPEC-J2 by WB. **(C)** mRNA expression by RT-qPCR of jejunal tissue in mice. **(D)** The abundance of ND2 and COXII in mice by WB. *means *p* < 0.05, **means *p* < 0.01.

To assess the influence of OXPHOS-related genes on STa-stimulated IPEC-J2 cells, we performed RT-qPCR to examine the changes in ND2, COX2, ATP6, and CYTB. We observed a clear decrease in ND2, COX2, CYTB, and ATP6 in the *E. coli* O142-infected group (Figure 6A) compared with *E. coli* O142Δ*estA*-infected cells. This trend was also observed for ND2 and COX2 (Figure 6B). Similar results were obtained for the jejunum of suckling mice (Figures 6C,D).

### 3.4 STa promoted mitochondrial oxidative stress and DNA damage and induced mitochondrial dysfunction

**Figure 7:**
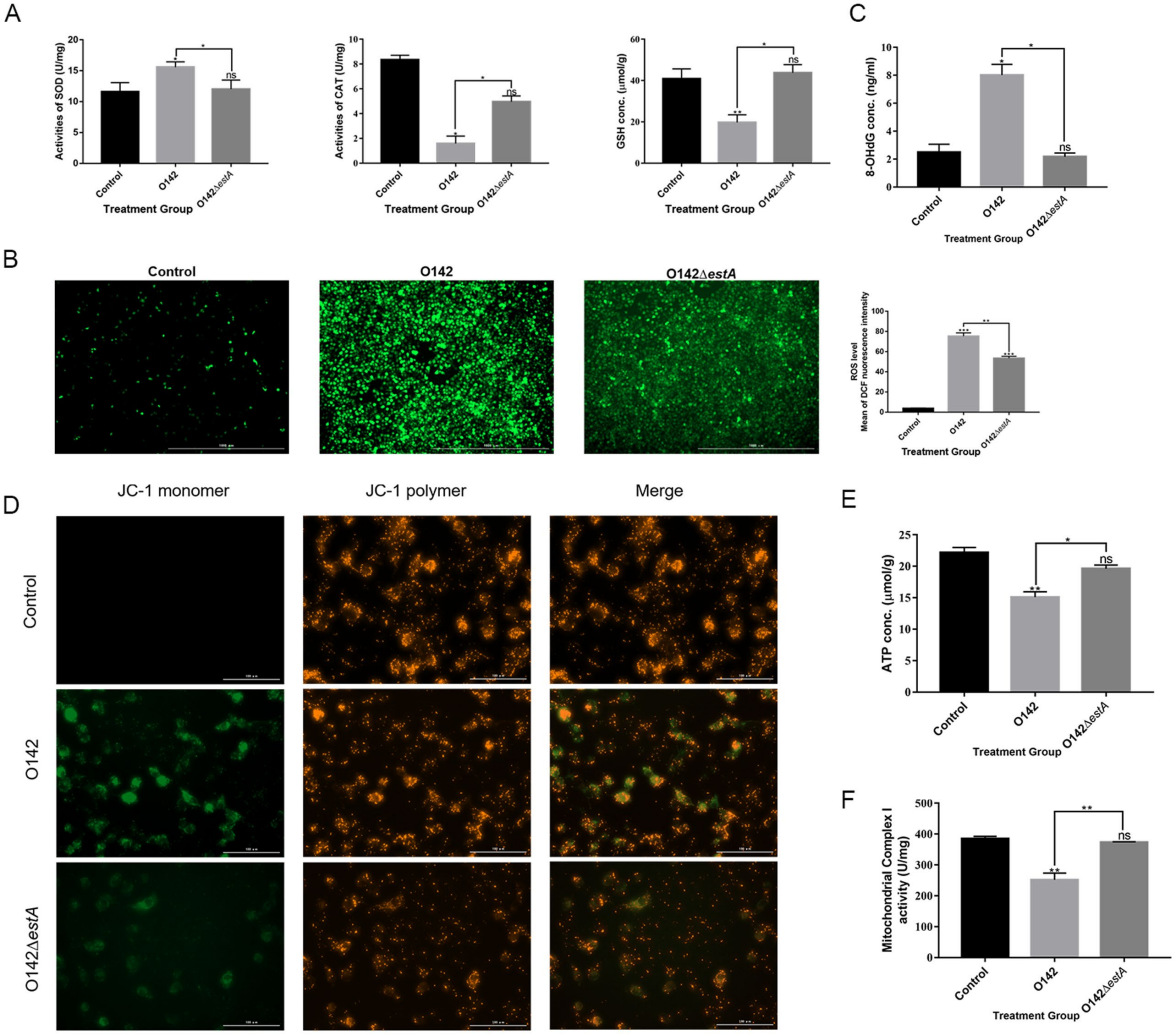
Effects of STa on oxidative damage in IPEC-J2 cells. **(A)** STa increased SOD and decreased GSH and CAT content in IPEC-J2 cells. **(B)** ROS fluorescence of IPEC-J2 cells. ROS generation was increased in IPEC-J2 cells by STa. Left is control group; middle is *E. coli* O142 infected group; right is *E. coli* O142Δ*estA* group. **(C)** Effects of STa on DNA damage in IPEC-J2 cells. STa escalated 8-OHdG level in IPEC-J2, causing DNA damage. **(D)** MMP in IPEC-J2 cells by JC-1. STa decreased MMP in IPEC-J2 cells. **(E)** ATP generation in IPEC-J2 cells increased after treatment with STa. **(F)** STa raised mitochondrial complex I activity. *means *p* < 0.05, **means *p* < 0.01.

The antioxidant system maintains the balance of redox reactions, and destruction of this system leads to oxidative stress in cells. SOD, CAT, and GSH are important components of the antioxidant system. Compared with the *E. coli* O142Δ*estA*-infected group, SOD content was increased in the *E. coli* O142-infected group, but CAT and GSH content decreased significantly (Figure 7A). A decreased GSH content is a significant marker of early apoptosis. The above results indicated that STa altered the antioxidant function of cells, and the instability of antioxidant function prevented cells from scavenging ROS over time. The stronger green fluorescence in the *E. coli* O142 group indicated that STa increased ROS levels (Figure 7B). If excess ROS cannot be removed in time, it will attack intracellular DNA, causing damage to cell structure and function, or even death. The effect of STa on DNA damage was investigated by measuring deoxyguanosine (8-OHdG) content. Compared with the *E. coli* O142Δ*estA* group, the 8-OHdG content in the *E. coli* O142 group increased significantly (Figure 7C), which proved that STa induced DNA damage in IPEC-J2 cells. These results suggest that STa causes oxidative damage to cells.

### 3.5 STa induces mitochondrial dysfunction

Mitochondrial complex I activity, MMP, and ATP levels were evaluated to examine whether STa induces mitochondrial dysfunction in IPEC-J2 cells. We observed increased green fluorescence in the *E. coli* O142 group compared with that in the *E. coli* O142Δ*estA* group, indicating that the MMP decreased (Figure 7D). Consistent with the effect of STa on the MMP, the production of ATP and mitochondrial complex I activity decreased significantly in the *E. coli* O142-infected group (Figures 7E,F). These results suggest that STa disrupts mitochondrial integrity and energy production.

**Figure 8:**
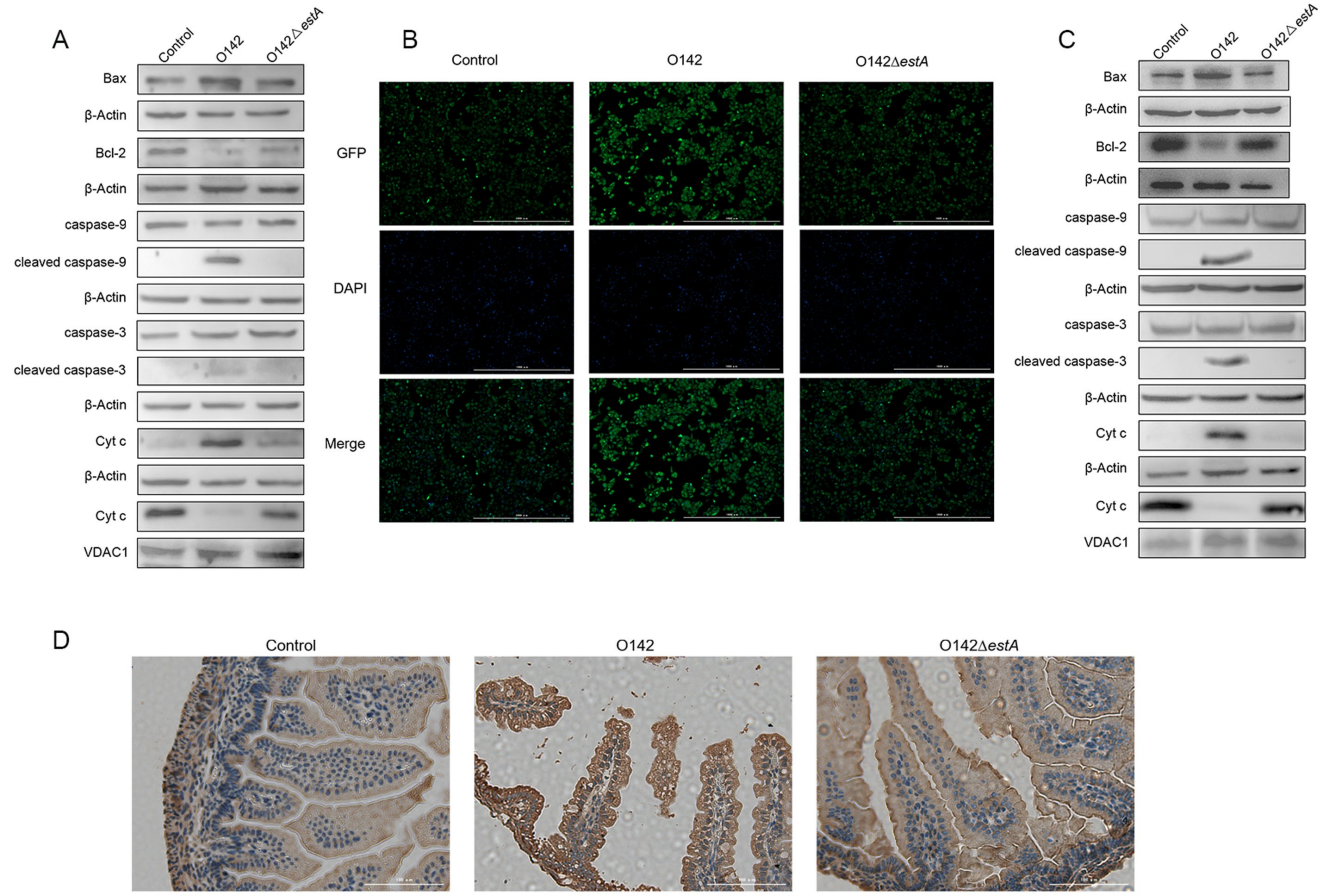
Effects of STa on the mitochondrial pathway of apoptosis. **(A)** The abundance of mitochondrial apoptosis pathway-related proteins in IPEC-J2 by WB. **(B)** The abundance of Cyt c in IPEC-J2 by immunofluorescence (IF). **(C)** The abundance of mitochondrial apoptosis pathway-related proteins in mice by WB. **(D)** The abundance of Cyt c in mice by immunohistochemistry (IHC). Three-day-old suckling mice were given 3 × 10^9^ *E. coli* O142 and *E. coli* O142Δ*estA* bacterial suspension, orally and, respectively. The negative control suckling mice were treated with the same dose of PBS.

We measured Bax protein expression in IPEC-J2 cells. The expression of the pro-apoptotic protein Bax was remarkably raised and that of the anti-apoptotic protein Bcl-2 was lowered in the *E. coli* O142-infected group, compared with the *E. coli* O142Δ*estA* -infected group (Figure 8A). The level of Cyt c in the mitochondria decreased, whereas in the cytoplasm, it showed the opposite trend in the *E. coli* O142-infected group (Figure 8A). Total CYTC protein levels increased (Figure 8B). These results suggest that the pro-apoptotic molecule Cyt c is released from the mitochondria into the cytoplasm, suggesting that STa-induced apoptosis is associated with the mitochondria. As a downstream molecule of Cyt c, the cleavage of caspase-9 in IPEC-J2 cells was significantly enhanced in the *E. coli* O142-infected group compared to the *E. coli* O142Δ*estA*-infected group. As a key executioner of apoptosis, caspase-3 was cleaved and activated in IPEC-J2 cells in the *E. coli* O142 group, but not in the *E. coli* O142Δ*estA* group (Figure 8A). The same results were observed in suckling mice jejunum tissue (Figures 8C,D). These results indicate that STa induces the mitochondrial apoptotic pathway.

## 4 Discussion

In this study, we investigated the mechanism of STa action on intestinal epithelial cells by comparing the differences after infection with *E. coli* O142 (STa^+^) or *E. coli* O142Δ*estA* (STa^−^) both *in vivo* (three-day-old suckling mice) and *in vitro* (IPEC-J2 cell line). Studies on the enterotoxins of ETEC could not be conducted in humans, and either suckling mice or piglets in an *in vivo* model were substituted. Studies on the enterotoxins of ETEC usually use suckling mice or piglets as *in vivo* model (17). STa shows biological activity in suckling mice and piglets (18). Zhou et al. (10) showed that STa produced the same effect on mouse and porcine enteroids *ex vivo*. The suckling mouse model is traditionally used for detecting STa activity (19–21). Schulz et al. (22) showed that 10^7^ CFU *E. coli* 1,676, which produced STa and expressed F41, caused sucking mouse death within 72 h after oral gavage. The results of STa-induced intestinal edema, diarrhea, and death were consistent with those of previous studies. Some studies have shown that mice show damage to the jejunal villi after treatment with STa, and that the damage caused by STa is dose dependent (10, 23). Our results showed that with an increase in the intragastric dose of *E. coli* O142, suckling mice gradually showed intestinal swelling and diarrhea symptoms, and the intestinal villi gradually atrophied and ruptured, all of which support the above views.

STa generally exerts its effect on the small intestine, particularly the jejunum. The IPEC-J2 cell line is an undifferentiated jejunal epithelial cell line. The *E. coli* O142 strain used in this study contains both K99 and F41 pili, and the ETEC strain expressing these two types of pili bound heavily to IPEC-J2 (24). Zhou et al. (25) used IPEC-J2 and *C. elegans* to study cell death caused by DH5α expressing STa, STb, and LT. Our study found that STa damages the cell wall and increases LDH secretion, which proves STa cytotoxicity. Annexin V-FITC/PI staining further indicated that STa increased the apoptosis of IPEC-J2 cells.

Apoptosis regulates the pathogenesis of several infectious diseases (26). Goncalves et al. (27) used the JC-1 probe to characterize NIH-3T3 cells after STb treatment and found that red fluorescence increased, which indicated that MMP decreased and mitochondrial-dependent cell apoptosis increased. RNA-seq data showed that STa significantly decreased the expression of mitochondrial respiratory chain-related genes and downregulated the OXPHOS signaling pathway in IPEC-J2 epithelial cells. OXPHOS is performed by the mitochondrial respiratory chain located in the inner membrane to produce the energy molecule ATP. The mitochondrial respiratory chain consists of five polymeric protein complexes (mitochondrial respiratory chain membrane protein complexes I-V), ubiquinone, and cytochrome C. Complex I is encoded by at least 37 nuclear and seven mitochondrial genes (ND1, ND2, ND3, ND4, ND4L, ND5, and ND6) (28). Mitochondrial complex I performs the first step in OXPHOS by converting NADH to NAD^+^ to generate the MMP required for ATP production, and produces ROS as a by-product (29). Antioxidant enzymes, such as SOD, CAT, glutathione peroxidase (GPX), and GSH, can remove ROS and maintain cell homeostasis. Our study showed that STa reduced CAT and GSH activity and increased ROS levels and SOD activity. Although the increase in SOD activity helps scavenge superoxide radicals, it generates H_2_O_2_, and H_2_O_2_ and superoxide radicals can generate harmful hydroxyl radicals through the Fenton reaction (30).

Excess ROS can attack intracellular biological macromolecules, such as proteins, lipids, and DNA. DNA damage in IPEC-J2 cells was observed by comparing the level of 8-OHdG in *E. coli* O142 and O142Δ*estA* groups after Sta exposure, suggesting that DNA damage is likely to be one of the response mechanisms of IPEC-J2 cells to STa. However, ROS and apoptotic protein upregulation damage the mitochondrial membrane and alter membrane permeability, thereby decreasing the MMP (31). Krzymińska et al. (32) observed that the *Aeromonas* enterotoxin increased intracellular ROS in HT29 intestinal epithelial cells, leading to a loss of MMP and apoptosis of epithelial cells via mitochondrial depolarization. Our results showed that STa induced ROS to disrupt MMP and lower ATP production, significantly increased the pro-apoptotic protein Bax, and decreased the anti-apoptotic protein Bcl-2. Changes in Bax and Bcl-2 lead to mitochondrial membrane permeability transition, causing nuclear translocation of Cyt c and its release into the cytoplasm, thus increasing cleaved caspase-9/caspase-9 and cleaved caspase-3/caspase-3, suggesting that STa initiates apoptosis via mitochondrial OXPHOS signaling pathways. T-2 toxin (1 and 2 ng/mL) also reduced MMP, induced Cyt c escape, caspase-3 and caspase-9 activation, and increased the Bax/Bcl-2 ratio after incubating mouse embryonic stem cells for 24 h (33).

Although in this study it was found that STa can activate apoptosis both *in vivo* and *in vitro*, and previous bacterial sequencing indicated that STa is the only known toxin component in ETEC O142 bacteria, due to the complex composition of bacteria and their metabolites and secreted substances, it is highly necessary to rule out the influence of other components of the bacterial cells on intestinal epithelial cells. It is essential to use purified STa to infect intestinal epithelial cells.

## 5 Conclusion

In conclusion, we demonstrated STa toxicity in intestinal epithelial cells *in vitro* and *in vivo*. We demonstrated that STa cytotoxicity was associated with excessive ROS production. Increased ROS attacks the mitochondria, leading to DNA damage and a decline in ATP, MMP, and Complex I activity. Changes in MMP lead to the release of apoptosis-inducing factors that activate the mitochondrial-mediated apoptosis pathway. Apoptosis of epithelial cells led to the destruction of intestinal epithelial integrity; thus, STa induced small intestinal edema, diarrhea, atrophy, rupture of small intestinal villi, and cell death in a dose-dependent manner.

## Data Availability

The original contributions presented in the study are publicly available. These data can be found in CNGBdb repository, accession number CNP0007299.

## Footnotes

^1^ https://github.com/tanghaibao/GOatools

^2^ http://www.genome.jp/kegg/

## Glossary

ETEC: Enterotoxigenic *Escherichia coli*
OXPHOS: Oxidative phosphorylation
LT: Heat-labile enterotoxins
GC-C: Guanylate cyclase C
CFTR: Cystic fibrosis transmembrane conductance regulators
CPE: *Clostridium perfringens* enterotoxin
SNAP23: Synaptosomal-associated protein 23
LB: Luria-Bertani
CFU: Colony-forming units
LDH: Lactate dehydrogenase
PI: Propidium iodine
HE: Hematoxylin-eosin
GO: Gene Ontology
KEGG: Kyoto Encyclopedia of Genes and Genomes
SDS-PAGE: Sodium dodecyl sulfate-polyacrylamide gel electrophoresis
TBST: Tris-buffered saline-0.1% Tween 20
VDAC1: Voltage dependent anion channel protein 1
Bax: Bcl-2 associated X
DCFH-DA: 2′,7′-Dichlorofluorescin diacetate
CAT: Catalase
8-OHDG: 8-Hydroxy-2-deoxyguanosine
IF: Immunofluorescence
IHC: Immunohistochemistry
BP: Biological process
MF: Molecular function
COX2: Cytochrome C oxidase
ATP6: ATP synthase
STa: Heat-stable enterotoxins
ROS: Reactive oxygen species
ST: Heat-stable enterotoxins
cGMPKII: cGMP-dependent protein kinase II
LT-IIc: Heat-labile enterotoxin IIc
Act: Cytotoxic enterotoxin of *Aeromonas*
ATP: Adenosine triphosphate
DMEM: Dulbecco’s modified Eagle’s medium
PBS: Phosphate buffered saline
MOI: Multiplicity of infection
TEM: Transmission electron microscopy
cDNA: Complementary deoxyribonucleic acid
DEGs: Differentially expressed genes
RIPA: Radio immunoprecipitation assay
NC: Nitrocellulose
RT: Room temperature
Bcl-2: B-cell lymphoma 2
Cyt c: Cytochrome C
SOD: Superoxide dismutase
GSH: Glutathione
ΔΨm, MMP: Mitochondrial membrane potential
DAPI: 4′,6-Diamidino-2-phenylindole dihydrochloride
*p*adj: Adjusted *p*-value
CC: Cellular component
ND2: NADH dehydrogenase
CYTB: Cytochrome B
GPX: Glutathione peroxidase
